# Engaging with the claim of Roma people through twitter: who is behind the hashtags?

**DOI:** 10.3389/fsoc.2023.1230954

**Published:** 2023-10-20

**Authors:** Emilia Aiello-Cabrera, Maria Troya, Ainhoa Flecha, Andrea Khalfaoui

**Affiliations:** ^1^Autonomous University of Madrid, Madrid, Spain; ^2^Autonomous University of Barcelona, Barcelona, Spain; ^3^University of Deusto, Bilbao, Basque Country, Spain

**Keywords:** Roma people, social media, twitter, hashtag activism, organizing, #RomaLivesMatter

## Abstract

Grassroots Roma communities play a pivotal role in organizing at the grassroots level, advocating for their rights, and challenging anti-Roma sentiment across Europe. Nevertheless, there remains a need for a deeper understanding of how these efforts manifest within the digital landscape. Within the overarching framework of the Narratives4Change project (EU Marie Curie Action, Nr. 841,355), this study seeks to examine the use of Twitter as a platform for advocating Roma-related issues. Specifically, it investigates the types of actors engaged in tweeting about Roma-related content and explores potential variations in profiles (organizations vs. individuals) based on the content being tweeted. The analysis encompasses six Roma-related hashtags spanning the years 2017 to 2020: #RomaLivesMatter, #InternationalRomaDay, #OpreRoma, #OpreRomnia, #MujerGitana, and #RomaWomen. The findings reveal that organizations are most active when employing the hashtags #InternationalRomaDay and #MujerGitana, whereas for the other hashtags studied, individual users dominate. Our data underscore the untapped potential of social media spaces and their ecosystems as strategic tools for advocacy and mobilization in support of Roma rights throughout Europe.

## Introduction

In November 2021, heartbreaking news was reported across various media channels and on social media networks: the tragic death of an 8-year-old girl in Greece, crushed by a sliding factory door. What compounded the tragedy was the indifference displayed by six or seven factory workers who passed by without intervening (Keep Talking Greece, 2021). This horrifying event joins a distressing list of cases involving individuals of Roma origin who have perished in situations tinged with antigypsyism (Council of Europe, 2012). This form of discrimination manifests either directly through racially motivated violence or indirectly when witnesses fail to intervene in acts of violence against the Roma community. The Roma community, Europe’s largest non-migrant ethnic minority and one of the most discriminated against (European Union, 2019), has organized. The Roma have harnessed the power of social media to expose ongoing instances of antigypsyism across European nations, advocating for the respect of their human rights (Vlase and Voicu, 2014; Schafft and Ferkovics, 2018).

Research has delved into how Roma people are leveraging social media to unite and raise their voices against social injustice and Antigypsyism (Nagy, 2018). Roma organizations and individuals have effectively employed platforms like Twitter and Facebook to provide aid and support to their communities at grassroots levels (Mercea, 2022). However, while it is evident that individuals of Roma background are utilizing social media for advocacy, comprehensive insights into key aspects of the Roma advocacy landscape, such as the most active participants and specific topics of community engagement, remain scarce. Unlike extensive research on the use of social media for protest and anti-discrimination efforts by ethnic minorities like African-Americans (Ince et al., 2017; Mundt et al., 2018), Latino communities (Zepeda-Millán, 2016) in the United States, or the Kurds in Turkey and Iran (Aghapouri, 2020), the Roma community has received relatively limited scholarly attention. The reasons for this might be attributed to the intricate dynamics within the Romani movement organization (Sigona and Trehan, 2009) and the transnational nature of Roma cultural identity, which transcends the conventional sense of national belonging in Europe (Sordé et al., 2013).

In this context, this study aims to explore and map the advocacy efforts for Roma rights on Twitter, with a particular focus on the profiles of those tweeting about these issues. Two central research questions guide our investigation: First, *what kinds of actors are sharing Roma-related content on Twitter?* Second, and related to this, *do the profiles of these actors (organizations* vs. *individuals) vary depending on the content they tweet?* Our analysis starts from the premise that social media significantly influence the creation and shaping of discourses surrounding the Roma population. These discourses have tangible consequences, either challenging persistent stereotypes and biases or perpetuating them. Grassroots Roma individuals play a pivotal role in organizing and advocating for their rights, both locally and nationally, as well as at the European level. However, there is much to learn about how these efforts manifest in the online realm. Furthermore, as highlighted in existing academic literature, a deeper exploration of how collective action regarding Roma issues, led by the Roma themselves, unfolds on social media can help identify online contexts where ethnicity fosters social solidarity within the transnational Roma network (Nagy, 2018).

The article is structured into four main sections. First, we review the literature on the use of social media for discourse positioning and activism, with a specific focus on its role in addressing racism and xenophobia. We conclude this section by shedding light on Roma activism in Europe and its (limited) usage of social media for advocacy. Second, we elucidate the methodological framework of our study. Third, we present our primary findings, discussing them in relation to the hashtags analyzed and providing insights into the most active profile types (organizations and/or individuals) and the nature of the shared content. In the final section, we discuss the results comprehensively and offer concluding remarks, emphasizing how Roma actors themselves, as well as advocates working towards Roma rights, can leverage social media tools and spaces for more strategic and purposeful organizing.

## Literature review

### Discourse positioning: the role of social media for activism

The incessant participation of millions of users around the world in social media has made the virtual space and the social media tools a powerful instrument for discourse positioning (Kaplan and Haenlein, 2010). Platforms such as Twitter and Facebook have been used in public debates for creating, sharing and mobilizing public opinion (Van Dijck and Poell, 2013). For this reason, although Twitter began its popularity especially in the academic and journalistic fields, it has spread in recent years to organizations, social groups, movements and activists (Marwick and Boyd, 2011). Considering that Twitter allows greater interactivity between people, regardless of whether or not they are part of their networks of friends, and that this translates into an exponential reach of a message, social media platforms have facilitated social movements and users both a fertile ground and a powerful organizing tool in which to operate and use, giving place to new ways of cyberactivism (Langman, 2005; Pickard, 2006; Sandoval-Almazan and Gil-Garcia, 2014). Web 2.0 has become a perfect complement to social protests, empowering citizens with different tools to achieve their main objective, protest; thus, these new tools have somehow transformed the traditional street protests into cyberactivism (Sandoval-Almazan and Gil-Garcia, 2014).

The explosion of political revolts and social movements occurred between the 2000s and the 2010s have been triggered by people’s deep organizing through social media (Mundt et al., 2018). During the last decade, the world has observed how social media has enabled an effective form of citizen journalism (Khamis and Vaughn, 2011), as occurred during the protests that took place in the Arabic Spring (Alaimo, 2015), the Umbrella Movement (Chu, 2018), the Tunisian revolution (Breuer, 2012), or more recently, the Black Lives Matter movement (Ransby, 2018). In these and other similar cases, social media has provided forums for ordinary citizens to document and capture *in-situ* protests, acting as both the loudspeaker to report human rights violations, disseminate political claims taking place on the ground, or even show to the world evidence of police brutality (Yang, 2016; Maas et al., 2018). But, as Harvard professor of leadership and organizing Ganz (2011) explains and reminds, it is not the power of social media platforms and tools *per se*, but how they are used as a key resource by people to also organize within the online domain. Digital platforms have transformed the notion that has been related to the duty of the media, traditionally constituted to disseminate messages, granting a passive role to the audience. More and more, contemporary activists use these social media to position messages, narratives, ideas and, at the same time, interact and build transnational connections with each other (Van Dijck and Poell, 2013; Khazraee and Novak, 2018; Mundt et al., 2018).

As used for political activism by protesters in ongoing democratization processes and advocating for the end of dictatorial regimes (Bruns et al., 2013), for gender equality and the end of sexual harassment, such as in the Me Too Movement (Quan-Haase et al., 2021), or for the rights of LGBT people (Becker and Copeland, 2016), to mention some examples, social media networks and cyberactivism have been also central for the denounce of situations of human rights violations suffered by ethnic and cultural minorities worldwide. Particularly relevant was the role played by social media to denounce the situation of violence and abuse in the case of the Black Lives Matter movement, but also against the Kurds (Onbasi, 2015), the Muslim ethnic Rohingyas (Elysayed, 2020), or the rights violations suffered by refugees including women in their journey and arrival to Europe in recent years (Dekker et al., 2018).

### Social media activism to report situations of racism and xenophobia: the 2015 refugee crisis and #BlackLivesMatter

Civil society has emerged as a prominent actor in addressing the 2015 “refugee crisis,” largely through the utilization of social media. In 2015, a vast number of individuals sought refuge across the Mediterranean Sea to escape war and persecution. The tragic image of a Syrian boy who had drowned drew global attention to the dire situation of refugees, awakening the world to the Syrian refugee crisis. Behavioral data suggests that, in this instance, a single iconic photograph of a child carried more emotional weight than cold statistics, leading to heightened concern for the Syrian crisis among those previously unaffected by the rising death toll (Slovic et al., 2017). This photograph was shared millions of times online, triggering a shift in focus that sparked increased global engagement with the refugee issue. This photograph swiftly became an icon, akin to other powerful images of children in humanitarian crises. As some authors have noted (Sajir and Aouragh, 2019), the impact of this image-driven narrative stimulated new forms of activism and mobilized grassroots participation, underscoring the argument that social media incites commitment through emotional connections.

However, it is important to note, as previous research has cautioned, that while shocking images may evoke compassion for the oppressed, they do not always translate into effective acts of solidarity and can instead devolve into ineffective forms of pity (*Ibid.*). Given that social media is an open platform where anyone can post, share, and comment without traditional media oversight, it has also provided a platform for anti-immigration movements and racist discourses to gain ground. In this context, much of the existing literature has focused on how social media has been used to disseminate hate and racism against refugees and migrants. For example, studies have shown that while the anti-immigrant movement employs ironic political strategies across various levels, from discourse to trolling, solidarity movements tend to emphasize compassionate yet increasingly practical and guarded approaches, as well as commercialization (Nikunen, 2018). Therefore, social media has become a commercialized and contested space of participation, not only for progressive political activism but also for expressions of hostility and racism. For instance, a wide body of research has pointed out how anti-immigration and hate speech proliferated on platforms like YouTube during the 2015 refugee crisis (Laaksonen et al., 2020). Consequently, in many instances anti-immigration political messaging has been normalized, multiculturalism framed as a threat, and proponents of multiculturalism discredited (Nortio et al., 2021).

Nevertheless, as mentioned earlier, the sharing of images and content in solidarity serves a functional purpose in activism, as it sheds light on the suffering endured by the oppressed and spreads their message, enabling a broader audience to become aware of their plight (Sajir and Aouragh, 2019). Therefore, it is imperative to delve deeper into how activism generated on social networks can serve as a powerful tool for effecting change. In this context, the Black Lives Matter movement has been a subject of study in terms of how social media networks have become avenues for empowerment and social and political activism. Since 2012 in the United States, the hashtag #BlackLivesMatter has been consistently and exponentially used on social media platforms to document cases of violence and inequality stemming from racism and advocate for changes benefiting marginalized groups (Ince et al., 2017). Social media has served as a narrative amplification tool within the Black Lives Matter movement, enabling activists to control their own narrative and raise awareness of the issues they address (Mundt et al., 2018). Similarly, support for the Black Lives Matter movement has transcended borders, generations, racial backgrounds, and religious affiliations, offering a means to unite various oppressed populations and, in some instances, those who identify with Blackness specifically (Rucker-Chang, 2020). Similarly, analogous hashtags have emerged to organize media spaces advocating for Roma issues, including the hashtag #RomaLivesMatter, which is discussed later in this study.

In summary, while social media can be a potent platform for disseminating hate and anti-immigration discourses, it also provides an opportunity to amplify narratives of solidarity and empathy, thereby addressing two critical aspects of collective action: giving voice and visibility to the marginalized (Plaut, 2012). Ultimately, the challenge lies in harnessing the potential of social networks to facilitate dissonance for social change and solidarity.

### Roma activism in Europe: from historical struggles to contemporary challenges and online engagement

Since their arrival on the European continent between the 9th and 14th centuries from Northwest India, the Roma people have endured a series of discriminatory episodes that have relegated them to the fringes of European society. Even after more than five centuries of coexistence, the Roma in Europe are still perceived as outsiders, raising questions about whether they should enjoy the same rights as other European citizens (Borràs-Batalla and Oms-Graells, 2023). Consequently, a significant portion of the Roma population today faces daunting challenges related to poverty, low educational attainment, school segregation, labor market barriers, and poor health (Fundamental Rights Agency, 2023). Some authors have drawn parallels between the situation of the Roma community in Europe and that of Black people in the United States (Lane et al., 2014). Both communities share a common history of enduring systematic racism and marginalization by national states, which have often been complicit in their persecution (Ransby, 2018; Alexander, 2020). Furthermore, the media plays a detrimental role by perpetuating negative Roma stereotypes, contributing to prejudiced attitudes and discrimination (Breazu, 2022), a phenomenon also observed in the case of Black people in the United States (Jackson, 2021). These harmful representations of the Roma persist to this day, linking them to crime, violence, a lack of agency over their own lives, and portraying them as people perpetually in need of assistance (McGarry, 2014). The situation is particularly dire for Roma women, who often bear a triple burden: that of being women, lacking academic credentials, and being Roma (Aiello-Cabrera et al., 2022a).

Nevertheless, Roma communities have not remained passive in the face of persistent antigypsyism but have organized (Aiello-Cabrera et al., 2022b; Khalfaoui et al., 2023). Scholars agree that the origins of the Romani movement are closely tied to the unique political and social histories of each country or region. In Spain, the end of the Franco regime allowed the Roma to begin political organizing (Sordé, 2006), while in Germany, the first civil rights activists started organizing after World War II (Matras, 1998). In Central and Eastern European countries, the collapse of communist regimes, which brought more episodes of racism towards the Roma, led to increased mobilization within the Roma community (Mirga and Gheorghe, 1997). This way, throughout the 1990s and the new millennium, the Romani movement has become increasingly diverse, intersectional, and transnational (Vermeersch, 2017; Kóczé et al., 2019).

The concept of the “Roma nation” (Mirga and Gheorghe, 1997) or the Roma as a transterritorial people (Sordé et al., 2013) has been consistently discussed in relation to the Romani movement and Roma identity. This term, not used in the European Westphalian tradition, but rather as a way the Roma people describe themselves as a politically self-aware ethnic minority (McGarry, 2014), unites them to articulate their political and social demands (Sigona and Trehan, 2009; McGarry, 2014; Vermeersch, 2017). This transterritorial aspect of the Romani movement sets it apart, as it reflects both the ethnic and cultural foundations of the movement, bridging tradition with modernity. On one hand, Roma identity embraces traditional aspects of Roma culture, such as loyalty to extended family, respect for elders, and strong networks of solidarity (Mirga and Gheorghe, 1997; Sordé, 2006; Sordé et al., 2013). On the other hand, it advocates for the development of a modern Roma identity that celebrates diversity and equality within Roma communities, addressing the multifaceted challenges faced by the Roma. This is particularly relevant in the case of Roma women, who call for a Roma feminism that considers the intersections of gender, ethnicity, and class (Bitu and Vincze, 2012; Munté et al., 2020).

A significant portion of the academic literature addressing Roma politics and advocacy has predominantly centered on two key aspects: assessing the limited role that Roma have historically played in European politics and analyzing the hurdles they encounter while advocating for a Roma political agenda, especially at the European Union (EU) level (Vermeersch, 2017). Consequently, there remains a dearth of research that delves into the exploration of online political mobilization led by the Roma.

Existing literature concerning Roma issues in the context of social media has primarily fixated on one particular facet, namely, the utilization of social media as a battleground for combatting antigypsyism (Gavra, 2012; End, 2017; Chovanec, 2021). The hashtag #RomaLivesMatter has gained traction within social media platforms, serving as a rallying cry to protest and advocate for the rights of Roma communities (Costache, 2021). Hence, the #BlackLivesMatter movement has ushered in a transformative shift, amplifying the use of social media networks with a shared aim, that of ending racial and ethnic oppression not solely against Black people but against other traditionally discriminated groups, such as the Roma (Freelon et al., 2016). #BlackLivesMatter has thus become into an avenue to foster global solidarity against racism and various other forms of discrimination (Gaines, 2022). However, despite these online demonstrations against antigypsyism and the broader struggle to vindicate the rights of Roma people expressed on social media, a critical question arises: Can these advocacy dynamics effectively challenge and counteract discourses of hate and racism against the Roma? This includes the creation of an online space to foster solidarity and the potential for it to transcend the digital realm, similar to what was observed in the #BlackLivesMatter movement. Drawing on this, this study constitutes the first of a series of research articles focused on mapping and better understanding how the Roma social media space is shaped, its depth and amplitude, its actors, and its impacts.

## Materials and methods

This study is framed in the Narratives4change research project, which has received funding from the European Union’s Horizon 2020 research and innovation program, under the Marie Sklodowska-Curie grant agreement No. 841355. This is a 36 months research investigation developed at the Harvard Kennedy School (HKS) and at the Department of Sociology at the Universitat Autònoma de Barcelona (UAB, Barcelona, Spain). Two research questions guided the study. First, *which type of actors are twitting about Roma-related content?* Second and related to this, *are there differences in the profiles of those actors (organizations* vs. *individuals) depending on the content tweeted?*

The specific study underwent a thorough examination utilizing a two-phase approach that encompassed both quantitative and qualitative analyses. This research process encouraged an inclusive and egalitarian exchange among the co-authors, fostering discussions and achieving consensus on the study’s methodological framework (Roca et al., 2022). All co-authors participating in this study have extensive experience collaborating with grassroots Roma communities; additionally, one of them has a Roma background. In our scientific pursuits, this ongoing dialogue and collaboration with grassroots Roma communities across various European countries allow us to gain a nuanced understanding of the subject matter. In the case of this study, the continuous conversation revolved around crucial aspects related to its methodological design. This encompassed discussions on selecting the appropriate timeframe, identifying relevant hashtags for inclusion, categorizing different user types, and establishing a robust data analysis process. The team also considered the existing scientific literature on Roma issues and social media advocacy, as well as the lived experiences of Roma individuals whose stories and advocacy efforts were captured within the selected tweets for the study.

First of all, six hashtags related to the Roma advocacy were selected by the research team: #RomaLivesMatter, #InternationalRomaDay, #OpreRoma, #OpreRomnia, #MujerGitana and #RomaWomen. Once this was done, the first phase of the study, the quantitative analysis took place: all the tweets published within the 5 years period 2017–2020 which used the mentioned hashtags were identified, screened and selected, and codified. The second phase involved the qualitative analysis, which was done manually, and consisted of identifying and classifying the tweets according to the type of profile of the sender. Two categories were created: senders identified as *organizations*, and senders identified as *individuals*; in turn, sub-categories were created within these two, and all tweets were classified accordingly.

In the following sub-section, the data collection process is explained in detail.

### Data collection process

In pursuit of addressing our research inquiries, we established a comprehensive data collection process, comprised of six primary steps.

1. *Selection of social media source:* We chose Twitter as our primary data collection source. This social networking platform enjoys widespread recognition and is actively used by activists and organizations dedicated to advocating for the rights of the Roma community.
2. *Selection of hashtags:* the hashtag selection process was done dialogically among co-authors, considering our knowledge and experience organizing with the Roma. These chosen hashtags were required to encompass at least three key themes central to the Roma community’s concerns: ethnic issues and Anti-Gypsyism, topics associated with the affirmation of Roma cultural identity, and gender issues, with a particular emphasis on the activism led by Roma women. This criterion guided our identification of the most frequently used hashtags associated with Roma-related topics.

From the initial list of hashtags extracted, we specifically selected the four hashtags with the highest volume of tweets. These hashtags were: #InternationalRomaDay, #OpreRoma, #RomaWomen, and #RomaLivesMatter. Additionally, we included two hashtags that centered on Roma women’s issues and organizing and featured content published in Spanish to engage the Spanish-speaking audience: “MujerGitana” (Roma women) and “OpreRomnia” (Up Roma women!). Refer to Table 1 for descriptions of the selected hashtags.

1. Temporal period analyzed: The temporal period under analysis was determined following the selection of our target hashtags. We chose a span of 5 years, encompassing tweets from January 2017 to January 2021. This timeframe was deemed reasonable for obtaining a comprehensive overview of the subject matter. Opting for a longer period would have presented challenges in processing the vast amount of extracted data, while a shorter timeframe, such as 1 year, would have yielded insufficient data. It is worth noting that this 5 years duration applied to all hashtags except for one, namely #RomaLivesMatter. We were particularly interested in tracking the evolution of this hashtag in connection with the Black Lives Matter movement. The first time that #RomaLivesMatter was used was in 2015.
2. Manual dataset extraction: In the fourth stage of our study, we focused on extracting all the data from Twitter. While the Twitter API is a commonly used method its it has limitations such as the retrieval of only the last 7 days’ data and a limit of 100 tweets. To overcome these limitations, we opted for manual data extraction to compile all the tweets posted during the selected time period. Concurrently, we gathered additional information, including the number of retweets and user profile details such as the “biography”, “external links in the profile”, “number of tweets published by the user”, “number of followers”, and “number of accounts followed.” It’s worth noting that premium and enterprise levels of Twitter API access offer more extensive historical data retrieval capabilities, although our co-authors did not have access to these levels.
3. User categorization: Creating user profiles from unstructured and informal data can be a complex task. However, Twitter users can be analyzed based on the information they provide in their profiles, tweets, and overall behavior (Moeen Uddin et al., 2014). Furthermore, users can be categorized into distinct groups, including personal, professional, business, spam, and feed/news users. Given this framework, and in response to our first research question, we conducted a categorization of users. After an initial examination of identified user profiles, we classified them into two broad categories: (a) organizations, referring to associations of people regulated by shared objectives and (b) individuals, representing single human beings distinct from any group. Within each of these two categories, we further established subcategories. This subcategorization process was a collaborative effort among researchers and drew inspiration from existing works that have undertaken similar categorization tasks. See Table 2 for a description of the created subcategories.
4. First phase and second phase analysis: In the initial phase, a total of 1,098 tweets were manually extracted and classified in accordance with the categorization framework established in the previous stage. Using this dataset, we conducted a descriptive qualitative analysis, leveraging information from retweets and user followers. For the second phase of our analysis, we employed NVivo software (Version 12). In this stage, our aim was to identify additional hashtags that appeared in all tweets extracted during the first phase. These tweets were those that included any of the six hashtags under examination and were selected for inclusion in our study. NVivo was also utilized for the qualitative analysis of the tweet content.

**Table 1:** Hashtags’ description.

#OpreRoma	In English, “Up, Roma” is a motto employed by Roma individuals and organizations worldwide, signifying the enduring spirit of struggle and resistance within a historically persecuted community.
#InternationalRomaDay	On April 8th, the International Day of Roma People is commemorated—a day dedicated to celebrating Roma culture and advocating for awareness of the challenges confronting the Roma community. Each year on this occasion, the hashtag #InternationalRomaDay is employed by numerous organizations and activists who highlight the ongoing issues of social exclusion and racism that many Roma individuals still confront in Europe and worldwide.
#RomaLivesMatter	One year following the tragic murder of Trayvon Martin in 2012 in the United States and the subsequent court verdict in favor of the perpetrator, Black Lives Matter emerged as a movement advocating for change in America, championing the rights and dignified existence of the African American population. In subsequent years, the movement gained global recognition as similar incidents occurred, along with reports of racial-based abuses. It was in this context that the hashtag #RomaLivesMatter emerged, aiming to raise awareness and highlight the enduring struggle of the Roma people throughout history.
#OpreRomnia #RomaWomen #MujerGitana	These are three of the hashtags used to represent the resilience and activism led by Roma women. Throughout history, Roma women have united in solidarity to enhance their living conditions and advocate for their rights, as well as those of their community, without compromising their cultural identity (Aiello-Cabrera et al., 2019). Nevertheless, it has taken a considerable amount of time for mainstream feminism and academic discourse to acknowledge grassroots Roma women as equal participants in public discourse and the public sphere [62]. Given this context, we aimed to investigate how the social media conversation surrounding “Roma women” is evolving—specifically, who is engaging in these discussions and in what manner. The most widely used hashtag concerning Roma women’s issues is #Romawomen, which reflects the advocacy and organizing efforts of Roma women on an international scale. As mentioned earlier, we also sought to explore how the social media dialogue is shaping up within the Spanish-speaking audience. Hence, we included “MujerGitana” and “OpreRomnia,” which translates to “Up Roma women” in Romano (the Roma language). “Opre Romnia” became the rallying cry of grassroots Roma women who organized around the 1st and 2nd Conference of Roma Women, titled “The Other Women,” led by the Spanish Roma Women Association Drom Kotar Mestipen in 2010 and 2018, respectively.

**Table 2:** Description of subcategories for type of organizations and type of individuals.

Organizations		
Subcategory	Description	Example
Official entities	Sometimes referred to as bureaucratic structures, encompass government organizations, government campaigns and programs, as well as state associations. This category encompasses all such formal institutions and bodies associated with the government and its functions.	The European Union program for education, youth, training and sport (@EUErasmusPlus); Cross-party group of Members of the European Parliament (@ARDIEuroParl).
For-profit	It comprises organizations that provide products or services in exchange for payment. In these entities, the business owner generates income from the for-profit venture and may also distribute profits to shareholders and investors.	Alacran Entertainment, a fresh multi-media group, registered in the US Patent and Trademark Office (@alacrangroup); a digital consulting called Salesforce Partner & Digital Transformation Consultants (@begitalis) and more.
Non-profit	It pertains to a legal entity established for the collective or social good, where any surplus revenues beyond expenses are dedicated to furthering the organization’s mission. This category primarily encompasses grassroots organizations, non-governmental organizations (NGOs), and non-profit foundations, all of which operate with a primary focus on social or community benefit rather than generating profits for individuals or shareholders.	@Romanipe, an organization whose main mission is to defend the dignity and human rights of Romani People worldwide; @DromKotar, the “Roma Association of Women Drom Kotar Mestipen,” based in Spain
Media	Refers to the organizations that inform and communicate to the population about issues of social relevance or situation.	@TFeminista, a feminist magazine in Spain @InannaPub, is one of the independent feminist presses remaining in Canada and is run exclusively by women.
Academic or research institutions	Refer to educational establishments primarily dedicated to both education and research. These institutions may include universities, as well as public or private research centers that are closely affiliated with universities. Their primary focus is on advancing knowledge through academic research and providing educational opportunities to students.	Cardenal Herrera University (@uchceu); Roma Peoples Project at Columbia University (@RomaPplsProject)

Individuals		
Subcategorization	Description	Example
Policymakers	“Policymakers” are users who have a direct connection to the political sphere and explicitly state this affiliation in their user profile description. This category encompasses individuals such as deputies, assembly members, parliamentarians, diplomats, ambassadors, and others who hold positions of authority and responsibility within the political arena.	Chief of the Organization for Security and Co-operation in Europe, the head of Political & Public Affairs, Embassy of Canada to Ukraine, and more. Roma activist, Member of the European Parliament
Public individuals (different than policy-makers)	“Public individuals (different than policy-makers)” refer to users who have achieved significant influence or recognition for reasons unrelated to politics. In determining this category, factors such as the information provided in their biography and the number of followers on their Twitter profile were considered. Specifically, users with more than 1,000 followers were categorized as “Public individuals” based on their notable influence or recognition.	**Note:** In the case of organizations, to clarify about the classification performed, some examples of real users were exposed. However, to protect the identity of individuals no real examples of the type of profiles are provided.
Journalists	“Journalists” are individuals whose primary occupation involves writing or producing news content for various media outlets, including magazines, television, radio, digital and traditional newspapers, and other forms of media sources. In this study, they were identified by their self-declaration as journalists in their user profile. Often, they also included additional information and links to the media organizations they work for on their profiles.	
Activists and/or academics	The category of “Activists and/or academics” combines two seemingly distinct groups: activists and academics. Activists: This group consists of individuals who openly declare in their user profiles that they are activists working in the realm of Roma rights advocacy. These users are actively involved in advocating for the rights and well-being of the Roma community. Academics: This group includes individuals who identify themselves as academics in their user profiles and typically hold academic credentials and positions within educational institutions. Some of these academics also address Roma-related issues on Twitter, and in some cases, they may overlap with the activist category, indicating their dual roles in academia and advocacy for Roma rights.	
Domestic users	The category of “domestic users” (Moeen Uddin et al., 2014) encompasses individuals who do not fall into any of the previously mentioned categories. These users are typically not affiliated with any organization, and they tend to have a low-profile presence on Twitter, with limited interactions with other users. They are essentially regular, non-affiliated Twitter users who may engage with Roma-related content to varying degrees.	

### Description of data

Table 3 provides an overview of the total counts for tweets and users in the complete dataset, as well as for each individual hashtag. These counts represent the final sample used for our study and encompass retweets, which are original tweets that have been shared by other users on the platform.

**Table 3:** Sample data description.

	Tweets	Retweets	Total Nr of tweets and retweets	Nr of users
All	1,098	21,175	22,273	704
#OpreRoma	227	1,239	1,466	202
#InternationalRomaDay	394	11,427	11,821	269
#RomaLivesMatter	212	7,802	8,014	108
#OpreRomnia	38	187	225	18
#RomaWomen	145	346	491	63
#MujerGitana	82	174	256	44

Tweets can contain more than one hashtag and can therefore be included in more than one row.

### Limitation

There are two significant limitations tied to the methodological design of this study that warrant consideration.

First, it is important to acknowledge that, as with any categorization process, there is no one-size-fits-all model. In some instances, categorizing both organizations and individuals into specific sub-categories posed challenges. When faced with such ambiguity, researchers engaged in individual case discussions and collaboratively determined the appropriate sub-category allocation.

Second, to obtain tweets published within the specified temporal periods, manual extraction was employed. This choice was necessitated by the limitations of other tweet retrieval methods, such as Streaming, REST (GET search), and REST (GET statuses), which do not support historical searches on the scale required for this study. Manual extraction, however, carries limitations in terms of the type of information that can be obtained, as there is no comprehensive Twitter database at the time of user registration. Consequently, metrics used in the analysis do not encompass user gender, which is a relevant aspect when examining Twitter user profiles.

### Ethical issues

The Institutional Review Board (IRB) of the Harvard University-Area approved this study, IRB Registration Nr: IRB00000109. In addition, all information gathered for the Narratives4Change project complies with the Ethics Appraisal Procedure required by the Horizon 2020 research program, funded by the European Commission. Accordingly, Narratives4Change project follows the Regulation (EU) 2016/679, the EU new General Data Protection Regulation (GDPR).

## Results

In this section, we present and discuss the main findings of the Twitter hashtags. We analyze them based on their outreach, the user profiles of those who used them in their tweets, and the most frequently used hashtags that appeared alongside the hashtag under analysis. We will present and discuss the results for each of the six hashtags included in the analysis.

### Our data in numbers: outreach and type of profiles

**GRAPH 1:**
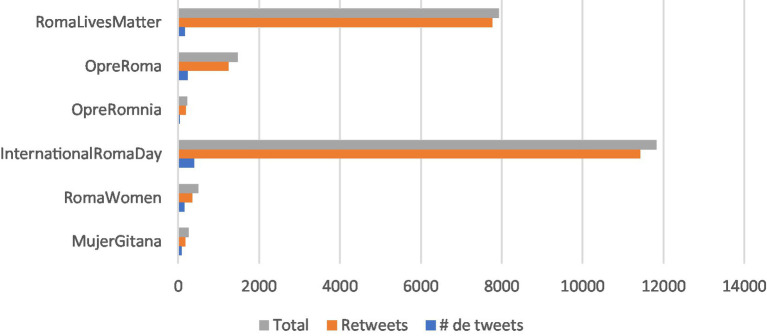
Hashtags outreach for the 5 years period (January 2017–January 2021).

In Table 3, as presented in the previous section (Data Description), we depict the outreach metrics for each of the six hashtags included in our analysis. As observed, #InternationalRomaDay exhibits the highest outreach, garnering 394 tweets and 11,427 retweets. Following closely, #RomaLivesMatter ranks as the second most influential hashtag, with 212 tweets and 7,802 retweets (also refer to Graph 1). There exists a significant disparity between these top two hashtags and the third one, #OpreRoma, which saw usage in 227 tweets and 1,239 retweets. Despite #OpreRoma being employed in more tweets (*n* = 227) compared to #RomaLivesMatter (*n* = 212), it did not receive as much sharing as the latter. On the other hand, #RomaWomen (*n* = 124), #MujerGitana (*n* = 82), and #OpreRomnia (*n* = 38) were the least utilized hashtags, with no significant differences among them.

The discussion of the study results will be guided by Tables 4–6, along with their corresponding graphs. Firstly, Table 4 provides insights into the types of user profiles associated with each of the six hashtags, distinguishing between individuals and organizations. It is worth noting that the number of analyzed tweets is not directly linked to the number of users for each hashtag, as some individuals and organizations have tweeted multiple times.

**Table 4:** Type of users’ profiles for each of the hashtags.

Hashtag	General / total count	Individuals		Organizations	
		Nr	%	Nr	%
#InternationalRomaDay	269	112	41.64	157	58.36
#OpreRoma	202	155	76.73	47	23.27
#RomaLivesMatter	108	90	83.33	18	16.67
#RomaWomen	63	33	52.38	30	47.62
#MujerGitana	44	17	38.64	27	61.36
#OpreRomnia	18	12	66.67	6	33.33

**Table 5:** Type of organizations users for hashtags (relative and absolute values).

	Organizations											
	Official		Media		Non-profit		Profit		Academia		Total	
	Nr	%	Nr	%	Nr	%	Nr	%	Nr	%	Nr	%
#InternationalRomaDay	61	39	9	6	82	52	2	1	3	2	157	100
#RomaLivesMatter	2	11	0	0	16	88	0	0	0	0	18	99
#OpreRoma	5	11	1	2	41	85	0	0	0	0	47	98
#OpreRomnia	1	17	0	0	5	83	0	0	0	0	6	100
#RomaWomen	12	40	1	4	15	50	1	3	1	3	30	100
#MujerGitana	7	26	3	11	15	55	1	4	1	4	27	100

**Table 6:** Type of individual users for hashtags (relative and absolute values).

	Individual											
	Policy-makers		Public individual		Activist/academic		Journalist		Other		Total	
	Nr	%	Nr	%	Nr	%	Nr	%	Nr	%	Nr	%
#InternationalRomaDay	39	35	2	2	39	35	8	7	24	21	112	100
#RomaLivesMatter	4	5	1	1	25	28	3	3	57	63	90	100
#OpreRoma	2	1	1	1	70	45	4	2	78	51	155	100
#OpreRomnia	1	8	0	0	6	50	1	8	4	34	12	100
#RomaWomen	2	6	0	0	20	61	3	9	8	24	33	100
#MujerGitana	0	0	0	0	8	47	2	12	7	41	17	100

**GRAPH 2:**
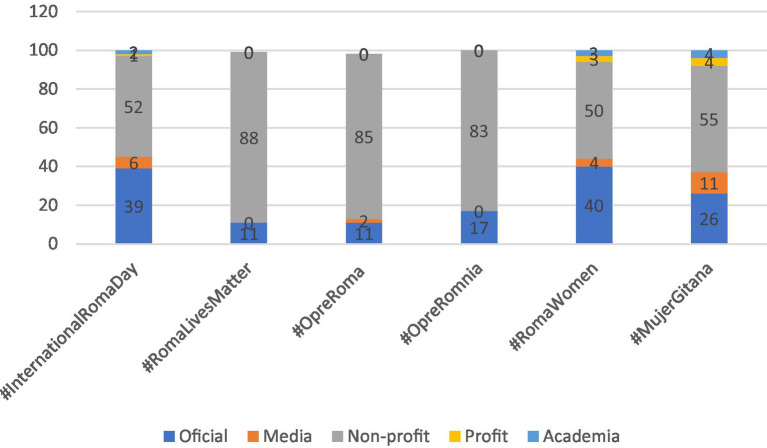
Type of organizations users for hashtags (%).

Next, Table 5, along with Graph 2, illustrates the distribution of the types of organizations utilizing each of the six hashtags. These organizations are categorized into five different types: official entities, for-profit organizations, non-profit organizations, media, and academic or research institutions.

**GRAPH 3:**
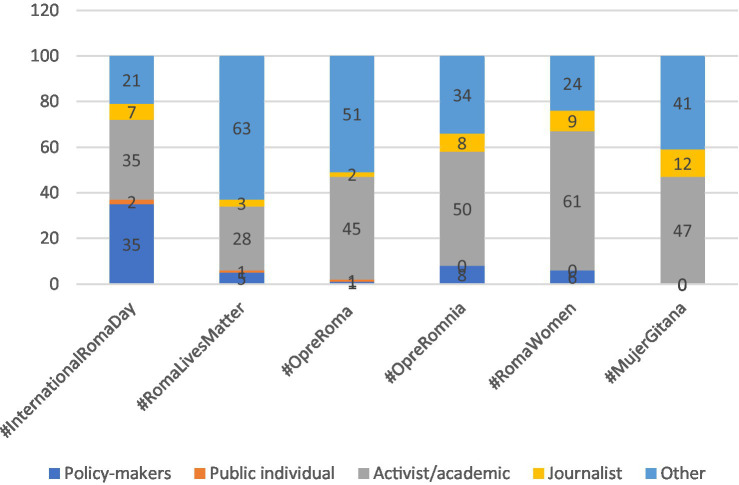
Type of individual users for hashtags (%).

Thirdly, Table 6, accompanied by Graph 3, presents the distribution of the types of individuals using each of the hashtags. These individuals are categorized into five different groups: policy-makers, public figures (distinct from policymakers, encompassing those with some form of influence), activists/academics, journalists, and *others*.

### Hashtag by hashtag: mapping the Roma social media space

In the following sections, we will present each of the analyzed hashtags, discussing the profiles of those using them—whether they are organizations or individuals—and the type of content they share.

#### #InternationalRomaDay: “#InternationalRomaDay, a day to celebrate Roma culture”

The utilization of the hashtag #InternationalRomaDay is relatively balanced between individuals and organizations. According to Table 4, 58% of the users are organizations (*n* = 157), while 42% are individuals (*n* = 112). Regarding the types of organizations employing this hashtag, the majority fall into two categories: 52% are non-profit organizations, and 39% are official organizations (as shown in Graph 2). Conversely, among individual users, policymakers and activists/academics each account for 35% of those participating in the hashtag’s usage, followed by Others at 21% (refer to Graph 3). Journalists and public figures have a limited presence in the utilization of this hashtag, at 7 and 2%, respectively.

**Figure 1:**
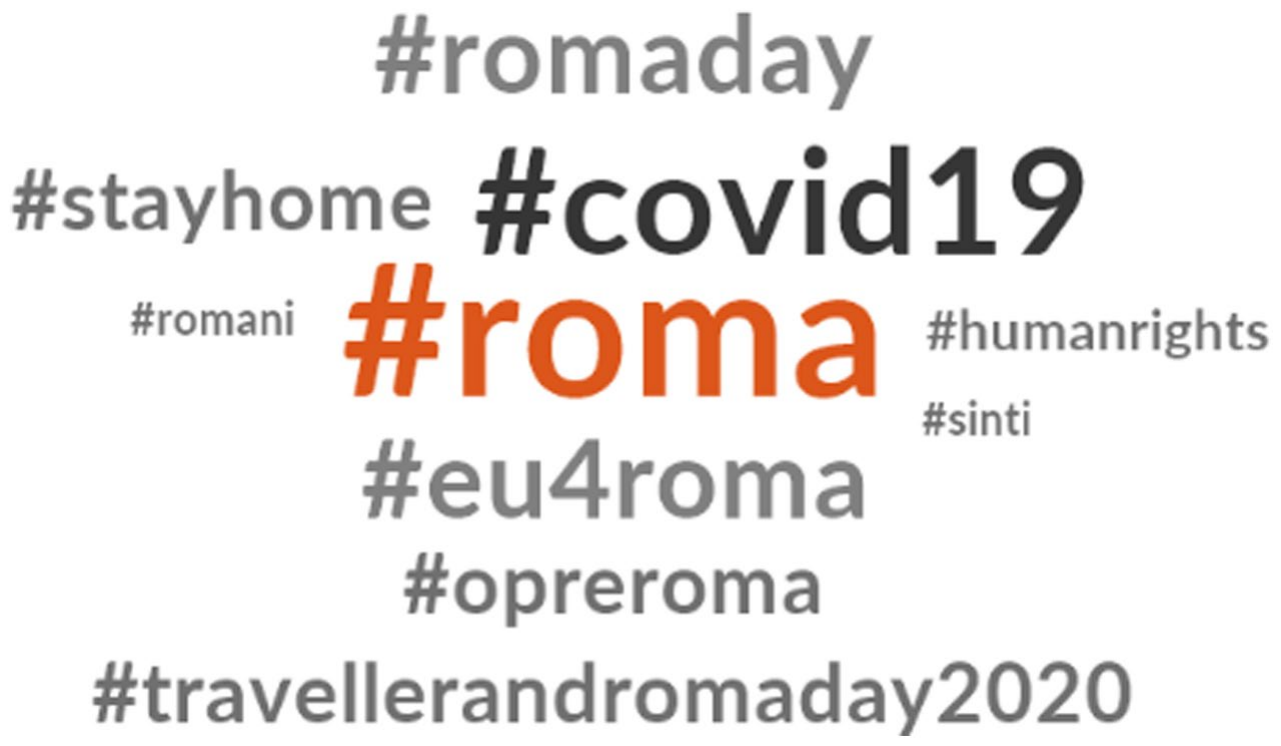
Hashtags related to #InternationalRomaDay.

Figure 1 presents the hashtags related to #InternationalRomaDay. Notably, #Roma and #covid19 are the most frequently used hashtags in tweets related to #InternationalRomaDay. These tweets often highlight existing inequalities exacerbated by the healthcare emergency. Additionally, there is repeated usage of hashtags related to European Union programs, such as #eu4roma. Some tweets incorporating #InternationalRomaDay also include the hashtag #Opreroma.

The analysis of #InternationalRomaDay reveals, as its name suggests, an international dimension. The findings indicate that this hashtag is not solely employed by the Roma community to commemorate their day of struggle (8th April) but also by policymakers and organizations seeking to convey a message of support to the Roma community:

Today is #InternationalRomaDay. It is a day to celebrate diversity in all its forms – culture, heritage, contribution to society, values, and ways of life. It is also a day to call for acceptance & social and economic inclusion of Roma and Egyptians. UNDP stands committed to this [User: official entity].

It can be inferred that this hashtag has become established for commemorating International Roma Day and disseminating information about programs designed to enhance the living conditions of the Roma community. This can be observed in the following tweet:

48% of Roma population in W Balkans has no access to health services. 50% of Roma women have no healthcare or pension coverage. In #Albania, Sidorela got her 1st job thanks to #EU – @UND initiative for #Roma social inclusion: http://bit.ly/2uhqEMy

#InternationalRomaDay [User: policymaker].

#### #Romalivesmatter: “Solidarity is beautiful! #Romalivesmatter”

**GRAPH 4:**
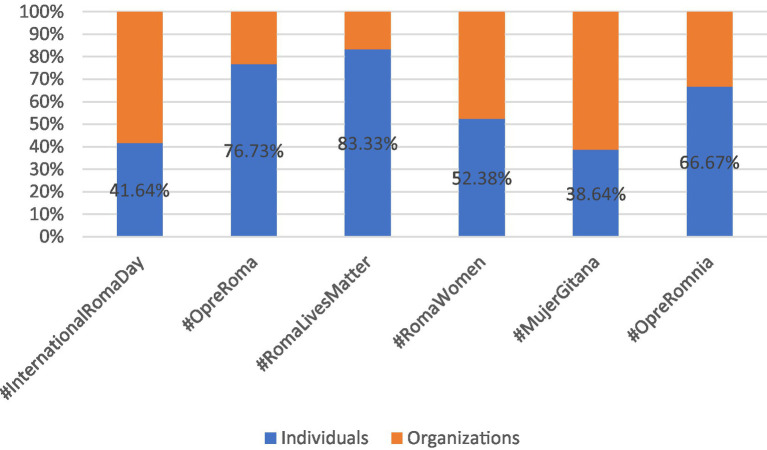
Types of users for hashtags (%).

In the case of #RomaLivesMatter, there is a notable disparity in the types of users employing this hashtag (as shown in Graph 4): 83% of those tweeting about #RomaLivesMatter are individuals (*n* = 90), while only 17% represent organizations (*n* = 18). Furthermore, as observed in Graph 3, individuals using this hashtag exhibit diverse profiles, with 28% identified as activists/academics and 63% categorized as “Others.” It’s worth noting that for this highly politicized hashtag, contributions from journalists, politicians, and public figures are limited, with none of these groups exceeding 5%.

Regarding the types of organizations using #RomaLivesMatter (refer to Table 5 and Graph 2), a pattern similar to that of #InternationalRomaDay emerges: 88% of them are non-profit organizations, and the remaining 11% represent official entities.

As expected for such a specific and politically charged hashtag, the data indicates a marked polarization in the distribution of profiles (individuals vs. organizations) using #RomaLivesMatter. Most of the users employing this hashtag are individuals, using it to raise awareness about rights violations and abuses against the Roma community, often drawing parallels with the #BlackLivesMatter movement in the United States. This may also suggest that organizations advocating for Roma rights are less engaged in framing the Roma issue as a clear and comparable struggle for racial justice, as seen in the case of #BlackLivesMatter.

**Figure 2:**
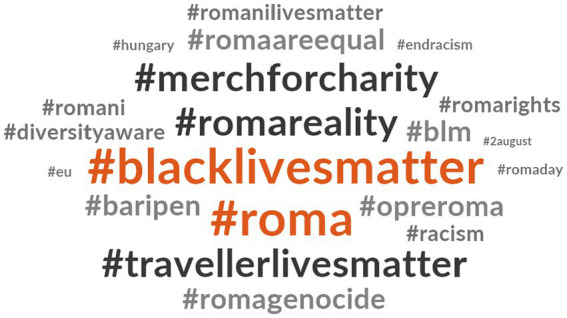
Hashtags related to #RomaLivesMatter.

Furthermore, the word cloud presented in Figure 2, depicting hashtags related to #RomaLivesMatter, highlights a distinct connection between this hashtag and #BlackLivesMatter. Other frequently used hashtags in association with #RomaLivesMatter include #Roma, #travellerlivesmatter, #merchforcharity, and #romareality. To a lesser extent, #opreroma appeared in some #RomaLivesMatter tweets, with two tweeted by organizations and six by individuals. Notably, all hashtags are in English except for #opreroma and #baripen, suggesting that #RomaLivesMatter is commonly used in English-speaking countries, while #OpreRoma is used in Spanish-speaking countries. Additionally, although less frequent, hashtags like #romagenocide and #2august were used, referring to Roma Genocide Remembrance Day.

The analysis indicates that #RomaLivesMatter serves as a platform created and primarily utilized by the Roma community and their grassroots movements. Below are some examples of tweets incorporating #RomaLivesMatter:

After #Ferguson and #NYPD, US in no position to preach, but racism in #France is truly remarkable: http://bbc.com/news/world-europe-30677067…. #RomaLivesMatter [User: academic/activist individual].

Young Romani male has been killed in the presence of Czech police #romalivesmatter #blacklivesmatter [User: public individual].

It is worth noting that, out of the total number of analyzed tweets, only one exhibited racist and stereotyped content. This particular tweet was retweeted twice, and the user had 18 followers.

Yesterday, a larger Roma group came to Palmovka to enrich us culturally. I have not seen such a mess left behind in the park under Koulí for a long time. So please @xxxxx about cleaning up, @xxxxxx about training how we behave in public and #romalivesmatter to come help clean up. #disgust [User: other individual] (Translated from Czech).

#### #Opreroma: “If we #Romani don't stand up for their lives, who will stand up for ours? #Opreroma”

Following a pattern similar to that of #InternationalRomaDay and #RomaLivesMatter, the hashtag #OpreRoma predominantly attracts individual users, accounting for 77% of those employing it (*n* = 155), with the remaining 23% representing organizations (*n* = 47) (as illustrated in Graph 2). Notably, users categorized as “Others” are prominent at 50%, and activists/academics make up 45% of those tweeting about #OpreRoma (as shown in Graph 3). Journalists, public figures, and policymakers collectively constitute the remaining 5%.

In a parallel to the #RomaLivesMatter trend, data reveals that 85% of the organizations using the #OpreRoma hashtag belong to non-profit organizations, while official organizations make up 11%, and media entities account for 2% (as indicated in Table 5 and Graph 2). Interestingly, there is no presence of for-profit organizations employing this hashtag. This can be attributed to the widespread recognition of the “Opre Roma” phrase and its significance within grassroots organizations, especially among grassroots women. This recognition stands in contrast to the more bureaucratic and formal terminology often used in official institutions such as European public institutions and similar entities.

**Figure 3:**
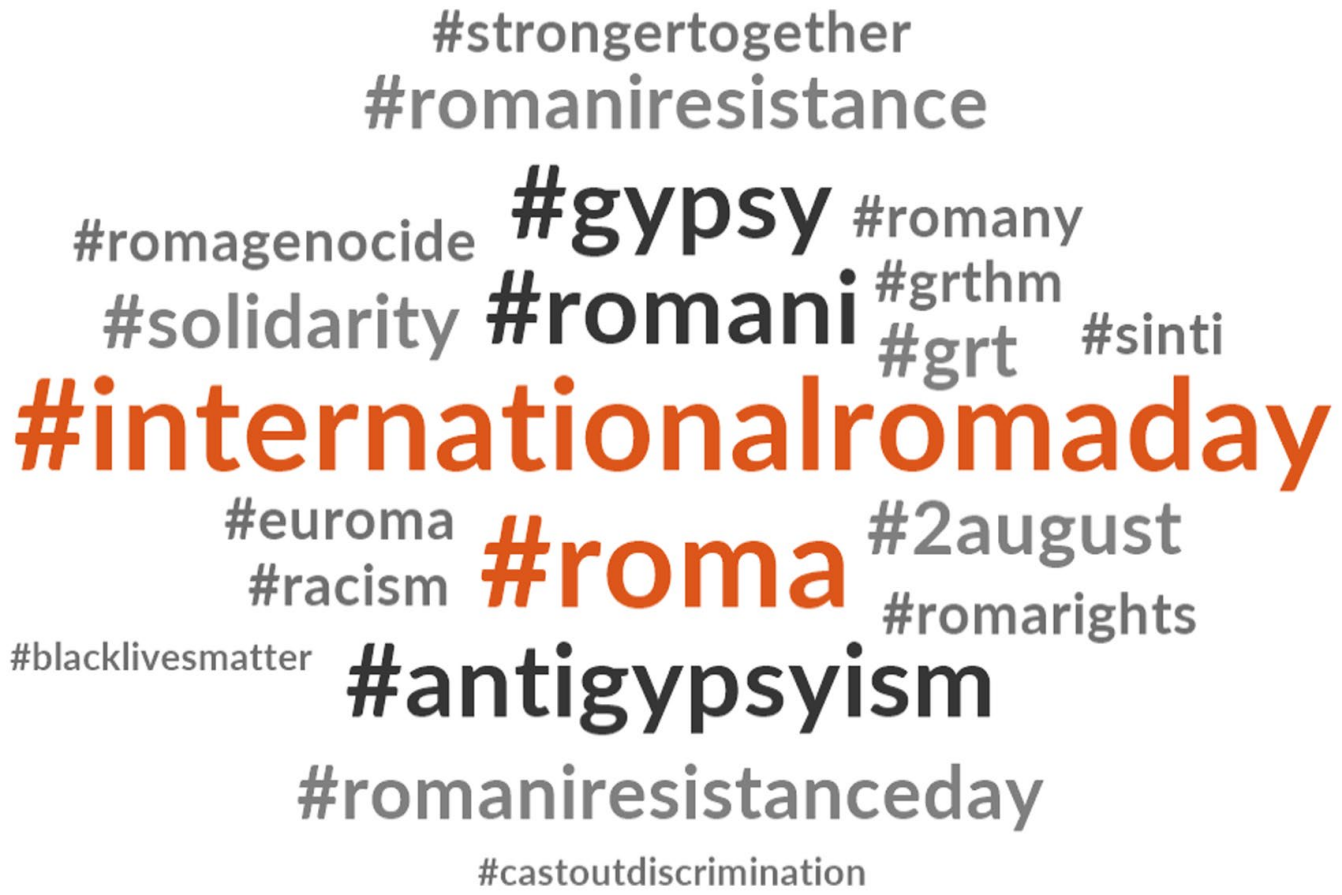
Hashtags related to #OpreRoma.

As depicted in Figure 3, the most frequently used hashtags in conjunction with #OpreRoma tweets include #internationalromaday and #roma. Additionally, the hashtag #antigysyism stands out, highlighting its association with tweets addressing situations of discrimination. These tweets often include other relevant hashtags such as #romaniresistanceday, #romarights, #romagenocide, and #solidarity, among others.

Considering that the majority of users in #OpreRoma tweets consist of non-profit organizations, individuals categorized as “Others,” and activists/academics, the purpose of this hashtag becomes evident, namely, to strengthen awareness of the Roma community’s advocacy:

Today marks an important moment. Today is the day Romani peoples and survivors in Canada and across the world are finally rightfully included in the history of #WWII. Today is a step towards dignity for Roma. #OpreRoma [User: non-profit organization].

Remembering the brave ones gone before, & those with us today – #GRT people in everyday life (mostly unknown or unreported), doing exceptional things for an equal & fair society, for the greater good of all. #RomaniResistanceDay #OpreRoma [User: *Other* individual].

As demonstrated in both of the examples provided above, the utilization of this hashtag encompasses messages centered on commemorating and empowering the Roma community in its quest to combat inequality and discrimination. Additionally, it's noteworthy to acknowledge that in the example below, the usage of the hashtag demonstrates a linkage to #BlackLivesMatter, fostering solidarity within the Roma community. Simultaneously, the first sentence of the tweet hints at a preexisting inquiry regarding the involvement of Roma individuals in the Black Lives Matter movement.

Although many people concerned about Roma joining #BlackLivesMatter protests, I will still join them until we Roma will be able to mobilize on streets and shout #OpreRoma or to raise social awareness on Roma issues as other activist groups do. SOLIDARITY! [User: *Other* individual].

#### #Opreromnia: “Tenemos voz, voto y hasta grito de guerra #OpreRomnia” (“We have a voice, a vote and even a rallying cry #OpreRomnia”)

Although the total number of tweets included in this analysis for #OpreRomnia is relatively low (*n* = 18, representing 2.5% of all analyzed tweets), the usage of this hashtag exhibits variations in terms of user profiles—individuals and organizations. Individuals make up the majority at 67% (*n* = 12), while organizations represent 33% (*n* = 6) (as depicted in Graph 4). Among the individuals using this hashtag, half are activists/academics, while a smaller percentage includes policymakers and journalists, each at 8% of the total.

Regarding organizations using the #OpreRomnia hashtag (as indicated in Table 5 and Graph 2), non-profit organizations play a predominant role, accounting for 83%, while official organizations make up a much smaller percentage, comprising 17% of the total attributed to organizations.

Interestingly, profiles affiliated with media and for-profit organizations did not employ the hashtag during the analyzed period. This data suggests that #OpreRomnia is primarily utilized by those directly engaged with grassroots Roma communities, advocating for and championing the cause of Roma women in Spain, and by those who understand the significance of the “Opre Romnia” motto.

**Figure 4:**
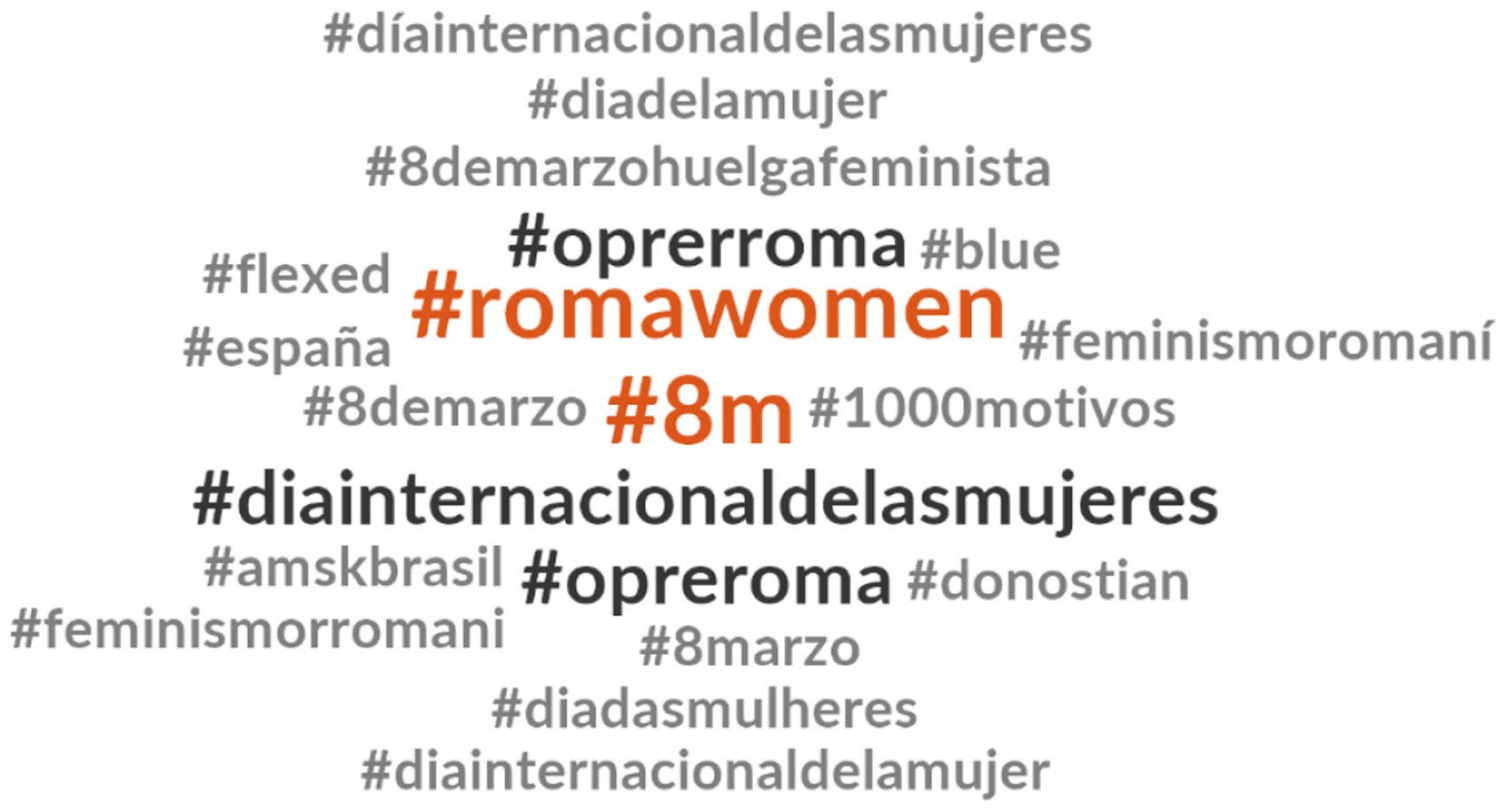
Hashtags related to #OpreRomnia.

In Figure 4, it’s evident that #romawomen and #8 m were the most frequently used hashtags in #OpreRomnia tweets. Additionally, #OpreRoma was frequently used in tweets containing #OpreRomnia. Many of the hashtags employed in the analyzed tweets pertain to the feminist struggle, including #8 m, #diainternacionaldelasmujeres, #8marzo, #diadasmulheres, #diainternacionaldelamujer, #feminismoromani, #8demarzohuelgafeminista, and #diadelamujer.

It is worth mentioning that, unlike the other hashtags we have analyzed, the use of the hashtag #OpreRomnia is primarily observed around March 8th, on Women’s Day. Below are some examples that capture the usage of #OpreRomnia:

For all those Roma women who walked the roads with our mothers in the ring! #OpreRomnia #DiaInternacionaldelasMujeres # 8ofMarzo [User: *Other* individual] (Translated from Spanish).

The connection to feminist movements is evident in the hashtags associated with #OpreRomnia:

This morning @levante_emv making visible the struggle of Roma women and other women’s groups for equality!! Feminism and Intersectionality!! @gitanos_org_CV [User: non-profit organization].

In all, those who tweet and share content using this hashtag tend to be individuals from academia and feminist activists:

We ended up the week sharing the story of our association with xxxxx, Prof. of the University of Educational Inclusion of @xxxxx, it was honour for us discussing with her how through egalitarian dialogue & educational actions is making huge steps #OpreRomnia [User: *Other* individual].

#### #Romawomen: “Keep on being visible #RomaWomen”

The total count of tweets identified under the hashtag #RomaWomen amounted to 145. From the analysis conducted, in contrast to the other hashtags under study, it is evident, as depicted in Table 4 and Graph 4, that there is a similar level of participation in the usage of this hashtag between individuals and organizations. Individuals contributed to 52% of all tweets captured with the #RomaWomen hashtag, while organizations accounted for the remaining 48%. Notably, activists/academics had a more significant involvement, comprising 62%.

Conversely, Graph 3 illustrates that journalists (9%) and policymakers (6%) had minimal participation in using the hashtag #RomaWomen, and no public individuals used it at all. On the other hand, Graph 2 shows that non-profit organizations (50%), as well as organizations designated as official entities (40%), played a significant role in employing the hashtag #RomaWomen. In contrast, the media (4%), for-profit organizations (3%), and academic or research institutions (3%) had a comparably small share in the overall usage, often refraining from using this hashtag.

**Figure 5:**
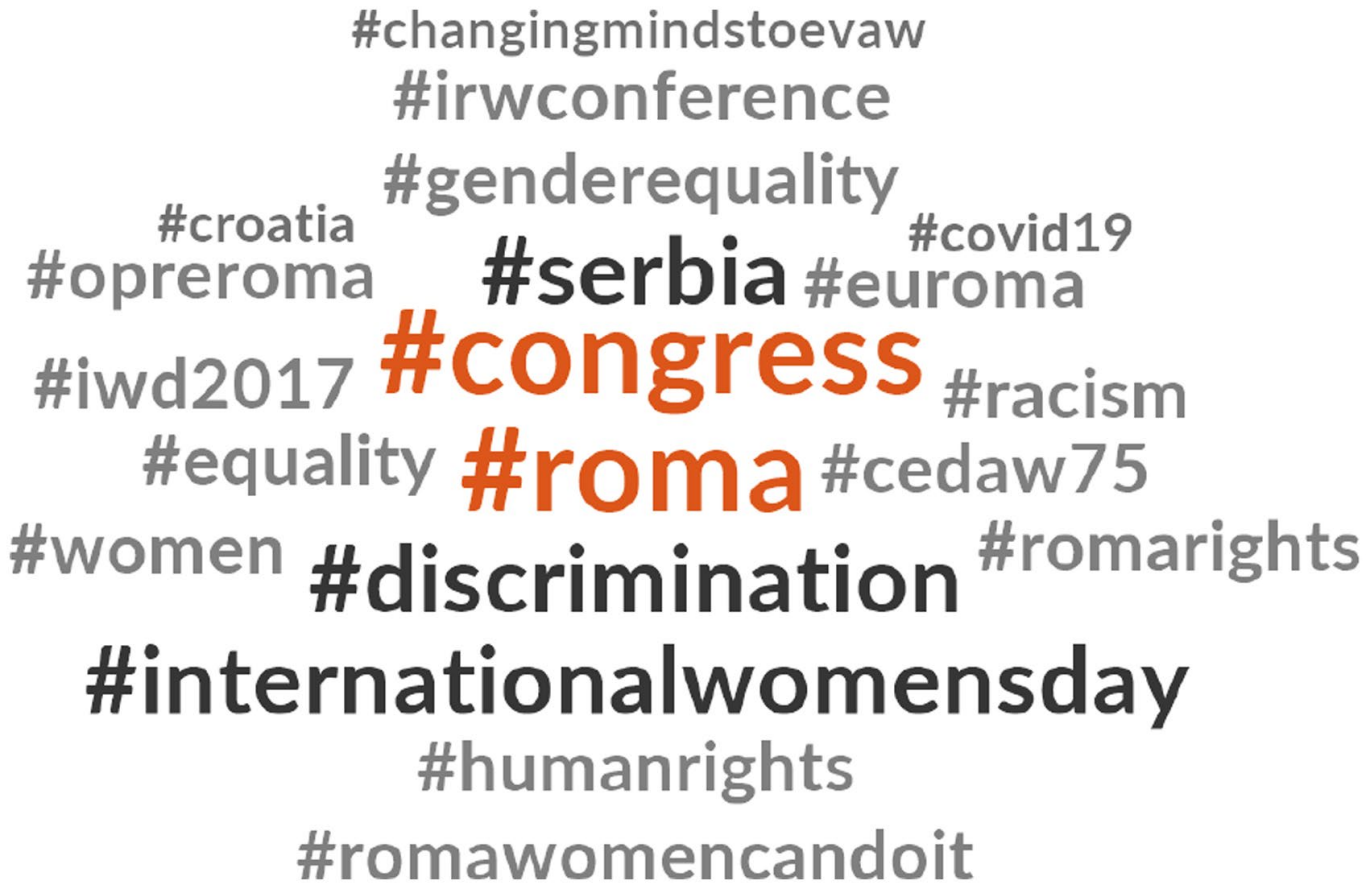
Hashtags related to #RomaWomen.

As depicted in Figure 5, the most frequently used hashtags in #RomaWomen tweets were #congreso and #roma. Additionally, #Serbia, #discrimination, and #internationalwomensday were utilized in some #RomaWomen tweets. Similar to previous cases, #opreroma accompanied some of the analyzed tweets. Much like the situation with #OpreRomnia, other hashtags that appeared in tweets containing #RomaWomen were those associated with the feminist struggle, such as #genderequality, #romawomencandoit, #equality, #women, or #cedaw75 (referring to the international Convention on the Elimination of All Forms of Forms of Discrimination against Women).

Similarly, as observed in the case of #OpreRomnia, the utilization of the hashtag #RomaWomen is primarily attributed to individual users, with a significant proportion falling under the activist/academic category:

Happy International Women’s Day https://youtu.be/pegMEihVMRc via @YouTube so proud to be a part of this campaign #InternationalWomenDay2020 #Romawomen #GypsyWomen #Travellerwomen [User: academic/activist].

In contrast to the prior scenario, our findings reveal a higher representation of official entities in the usage of this hashtag. Some of these organizations employ the hashtag to disseminate content aimed at increasing awareness about the enduring discrimination and racism experienced by the Roma community, with a particular emphasis on Roma women:

Equality Today in March focuses on: #AgeDiscrimination, #SDGs, #GoodLeadership, #ElectHope, #RomaWomen, #IWD2019, #FightRacism, #EqualityData [User: official entity].

However, when examining the subset of users represented by organizations who are actively tweeting using the #RomaWomen hashtag, it becomes apparent that the presence of official organizations is slightly less pronounced in comparison to non-profit organizations, activists/academics, and individuals. Below is an example of a tweet shared by a non-profit organization discussing #RomaWomen, in which they share a manifesto related to March 8th:

Read our statement on the upcoming occasion of the 8th March #InternationalWomensDay #RomaWomen = Generation #Equality. Realizing women’s rights for an equal future. https://gitanos.org/actualidad/archivo/130841.html.en… #GeneracionIgualdadMujeresGitana #8M2020FSG #GenerationEquality [User: non-profit organization].

While certain tweets and hashtags linked to #RomaWomen do make reference to the feminist movement, it’s important to note that this aspect is not as dominant as it is in the case of #OpreRomnia.

#### #Mujergitana: “En el día internacional de la mujer trabajadora, es de justicia acordarse de la #MujerGitana” (On International Day of working women, it is justice to remember the Roma women #MujerGitana)

#MujerGitana stands out as one of the less frequently utilized hashtags during the analyzed time period, with a total of 82 identified tweets originating from 44 users. This observation may be attributed to the fact that tweets featuring #MujerGitana are primarily in Spanish, and there is a smaller audience tweeting in Spanish regarding Roma-related issues when compared to those using English. Our data indicates that half of the users employing #MujerGitana are organizations, accounting for 61% of the total, while the remaining 39% are individuals with affiliations to universities, research groups, and non-profit organizations (as shown in Graph 4).

Regarding the profile of these individual users of the hashtag, 47% were identified as Activists/Academics, 12% as journalists, and the remaining 41% fell under the category labeled as Others (as depicted in Graph 3). It’s worth noting that #MujerGitana is not being utilized by policymakers in any capacity, which is an interesting observation. Below, you can find an example of a tweet using #MujerGitana, sent by an academic/activist:

Yes, there are publications about the tragedy experienced by these women: https://www.antropo…, https://adonay5… and https://unebook… #mujergitana [User: academic/activist] (Translated from Spanish).

Regarding organizations that engage with the #MujerGitana hashtag, more than half of them represent non-profit organizations (55%), followed by official entities, which constitute 26% of the total. The media accounts for 11%, while academic organizations and for-profit entities each comprise 4% (as indicated in Graph 2). In contrast to #OpreRomnia and #RomaWomen, where individuals were the primary users of the hashtag, #MujerGitana sees a higher level of engagement from organizations.

Furthermore, the use of this hashtag is predominantly observed when disseminating information about socio-educational initiatives targeted at Roma women, including workshops, documentaries, and various other social projects. Overall, it is evident that official Spanish organizations actively involved in addressing issues related to Roma women have a prominent presence within the virtual space shaped by the #MujerGitana hashtag.

¡MORE ACTIVITIES FOR TOMORROW! With the groups of WOMEN and EMPOWERMENT we are going to discuss what RESILENCE is. So important and essential. If someone wants to be part of it, contact us at: – Asociacion_romi@hotmail.com #QuedateEnCasa #mujergitana #laveugitana^1^ [User: non-profit Spanish organization].

**Figure 6:**
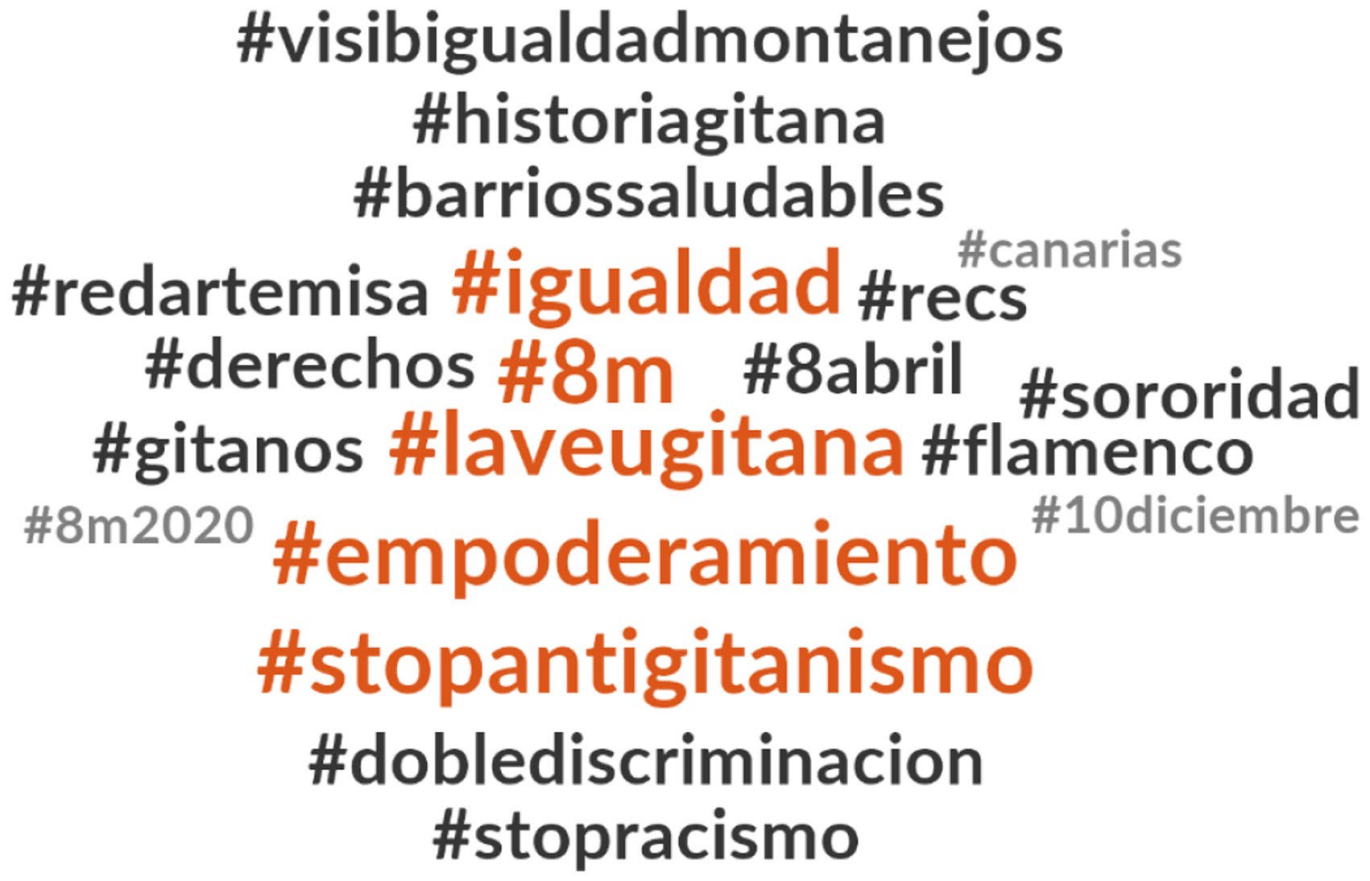
Hashtags related to #MujerGitana.

Our analysis of related hashtags reveals that, in the case of #MujerGitana tweets, the most frequently used hashtags include #igualdad, #8 m, #laveugitana, #empoderamiento, and #stopantigitanismo (as shown in Figure 6). These hashtags are all associated with the feminist struggle, promoting empowerment, equality, anti-racism, and International Women’s Day. Additionally, to a lesser extent, #sororidad (sorority) and #8 m2020 were also hashtags that appeared alongside #MujerGitana.

While Figure 6 does not display the hashtag #InternationalRomaDay, it is noteworthy that the hashtag #8deabril is observed, signifying the International Day of the Roma people. Furthermore, hashtags associated with Spanish civic and political organizations found use in #MujerGitana tweets, such as #redartemisa and #visibigualdadmontanejos. Additionally, #flamenco was employed as a symbol of Roma and Spanish culture. Although elements of remembrance, activism, and equality are evident in tweets featuring #MujerGitana (as exemplified in the tweet used as the section title, “On International Day of working women, it is justice to remember the Roma women #MujerGitana”), a significant portion of the discourse centers around socio-economic inclusion and anti-discrimination measures. This is particularly focused on addressing the dual discrimination Roma women face in the realms of education and employment. For instance, some of the most frequently associated hashtags in tweets containing #MujerGitana include #Barriossaludables (healthy neighborhoods), #derechos (rights), #doblediscrimination, and #Stopracismo. This emphasis is underscored by the substantial involvement of official entities in the use of the #MujerGitana hashtag, reflecting a policy-driven approach aimed at tackling the challenges encountered by Roma women in Spain.

In conclusion, the utilization of the #MujerGitana hashtag, predominantly by organizations and individuals within the Spanish-speaking community, signifies a concerted effort to address the multifaceted issues impacting Roma women. It is evident that #MujerGitana is intricately linked with broader feminist movements, advocating for empowerment, equality, and anti-racism, not only on International Women’s Day but also beyond.

## Discussion and conclusion

In this study, we conducted an examination of the engagement of various actors, including both organizations and individuals, in the creation, dissemination, and discussion of content related to Roma issues on Twitter. We mapped, categorized, and analyzed a total of 1,098 tweets featuring hashtags such as #InternationalRomaDay, #RomaLivesMatter, #OpreRoma, #OpreRomnia, #RomaWomen, and #MujerGitana (Roma Women in English), during a span of 5 years (2017–2021). Our findings unveil a diverse spectrum of profiles among those who tweet about Roma-related subjects using the aforementioned hashtags. This diversity appears to vary depending on the specific hashtag being analyzed. We noted that organizations predominantly use the hashtags #InternationalRomaDay and #MujerGitana (Roma Women in English). Conversely, for the remaining four hashtags analyzed, namely #RomaLivesMatter, #OpreRomnia, #RomaWomen, and #OpreRoma, the most active contributors tend to be individuals.

As occurred in the case of other social movements and social protests (Van Dijck and Poell, 2013; Yang, 2016; Mitchelstein et al., 2020), evidence collected suggest that the Roma social space is mostly being shaped in a very bottom-up way by individuals, and in a less extent but also of relevance, by non-profit organizations. Activists/academics (categorized in this study as “individuals”), play a pivotal role in discussions about the Roma community, actively engaging by sharing and commenting on related content. This group of users is particularly prominent in tweets featuring the hashtags #OpreRoma, #OpreRomnia, and #RomaWomen. Among users categorized as organizations, non-profit organizations are the most active in tweeting and sharing content concerning Roma issues.

Among all the hashtags analyzed, our findings highlight that #InternationalRomaDay and #RomaLivesMatter were the most widely circulated during the five-year period under study. This points to the international dimension of the Roma struggle and its connection to existing social and political protests. On the one hand, the popularity of #InternationalRomaDay is expected, as it signifies April 8th, an official day dedicated to celebrating Roma culture and raising awareness of the challenges faced by the Roma community. This day is not only observed by the Roma people but is also recognized by governments and international organizations working with the Roma community. Its use in English makes it accessible to users worldwide, underscoring the transterritorial nature of Roma cultural identity (Sordé et al., 2013).

On the other hand, the second most popular hashtag, #RomaLivesMatter, emerged in January 2015, two years after the rise of the #BlackLivesMatter movement. Notably, #RomaLivesMatter is often used alongside #BlackLivesMatter, primarily by individuals, as a gesture of solidarity with the Black Lives Matter movement while advocating for their own rights, equality, dignity, and respect. Some tweets have challenged the use of #RomaLivesMatter, arguing that “the Roma people have their own struggle,” and for this, #OpreRoma was already in use. However, the usage of #RomaLivesMatter following the emergence of #BlackLivesMatter points, as suggested by some literature, to the ability of the Black Lives Matter movement to inspire advocacy and activism not only for the rights of Black people but also for other ethnic and racial minorities who have long been victims of racism (Gaines, 2022). Despite the extensive scholarly attention that the #BlackLivesMatter movement has received, with thousands of scholars analyzing social movements and minority rights, both within and beyond the United States (Ince et al., 2017), there remains an absence of comparable analysis regarding the issue represented by “RomaLivesMatter.” While parallels can be drawn between the Roma people in Europe and Black Americans in the U.S. (Chang and Rucker-Chang, 2020), the political influence of the Black Lives Matter movement and its prominence on social media are undeniable, whereas the manifestation of the Roma movement in the social media space appears to be more limited. Moreover, while the Black Lives Matter movement has organized hundreds of disruptive protests in American cities since 2013, gaining considerable attention from the U.S. media (For instance, the killing of George Floyd in 2020 and its worldwide impact) (Bonilla and Tillery, 2020), situations of abuse against Roma people, like the episode of the 8-year-old Roma girl in Greece mentioned at the beginning of the study, although often reported, are far from receiving mainstream attention.

Hence, a future study could conduct a comprehensive comparison between Roma activism on social media in Europe and that of Black Americans in the U.S., exploring the explicit use of hashtags like #BlackLivesMatter and #RomaLivesMatter, assessing the reach of their networks, and gauging the attention they garner. While the current study does not specifically address this issue – and recognizing the demographic distinctions between the two groups, namely Roma and Black Americans – our data uncover that between 2015 and 2021, we identified 212 tweets featuring the hashtag #RomaLivesMatter, which spans a longer timeframe for this hashtag. This count may appear comparatively low when juxtaposed with the numerous instances of racism against Roma individuals that transpire monthly in Europe, as reported by the EU Fundamental Rights Agency (2023). One potential explanation for this disparity may be rooted in the persistent structural and systemic racism that continues to affect European societies and Roma communities. Consequently, there remains a limited response from civil society, whether through offline or online activism, whenever a racist attack against Roma individuals occurs and is reported.

With regards to the gender dimension, issues concerning Roma women are predominantly discussed through the hashtags #RomaWomen, #MujerGitana, and #OpreRomnia. Notably, non-profit organizations and activist/academic individuals play the most active roles as contributors. When comparing user engagement between #RomaWomen and #MujerGitana, it becomes evident that #RomaWomen is primarily utilized by individuals, whereas #MujerGitana is mainly embraced by non-profit organizations. This distinction can be attributed to the prevalence of grassroots organizations advocating for Roma women’s rights, particularly in Spanish-speaking countries. Furthermore, some tweets and hashtags associated with #RomaWomen touch upon feminist themes, although feminist discussions appear to be more pronounced under the hashtag #OpreRomnia.

Our previous research indicates that grassroots Roma women, often referred to as “Other women” (Flecha and Puigvert, 2010), who lack academic credentials and come from low socio-economic backgrounds, are organizing within their neighborhoods and communities, advocating for a unique form of Roma feminism (Sorde et al., 2013; Aiello-Cabrera et al., 2019). In many instances, they express not feeling represented by mainstream representations of White feminism. The data collected in this study suggests that the multifaceted expressions of Roma feminism led by Roma women’s organizations have yet to fully penetrate the online public discourse on Twitter. This suggests a need for further research to delve into the evolving landscape of Roma feminism in online environments and across various social networks. Such research would provide valuable insights into how Roma women and their allies leverage digital spaces to raise awareness and reach audiences beyond those already familiar with the Roma cause.

In all, the study reported in this article is of ground-breaking nature as it is the first one in exploring how the social media space in Twitter is being used to advocate for issues related with the Roma people. The struggle for the fulfilment of the Roma rights is also taking place in online settings, shaping and informing an organizing ecosystem of very diverse type of actors and organizations, that is, including Roma and non-Roma individuals, and Roma and non-Roma organizations which are working for the rights of the Roma.

Nonetheless, our findings suggest that the use of Twitter for Roma advocacy remains relatively limited. This underscores the potential for those engaged in Roma advocacy and activism to harness the online space as a strategic platform for building alliances and fostering broader awareness. Leveraging social media, advocates can address vital issues within the Roma community, including combatting antigypsyism, advocating for the realization of social and political rights, and addressing the specific needs of Roma women. Expanding their online presence and engagement offers an opportunity to amplify their voices, draw attention to pressing concerns, and mobilize support, ultimately contributing to greater awareness and positive change for the Roma community.

## Data availability statement

Data supporting the conclusions of this article will be made available by authors upon request.

## Ethics statement

The studies involving humans were approved by the Institutional Review Board (IRB) of the Harvard University-Area approved this study, IRB Registration Nr: IRB00000109. In addition, all information gathered for the Narratives4Change project complies with the Ethics Appraisal Procedure required by the Horizon 2020 research program, funded by the European Commission. Accordingly, Narratives4Change project follows the Regulation (EU) 2016/679, the EU new General Data Protection Regulation (GDPR). The studies were conducted in accordance with the local legislation and institutional requirements. The ethics committee/institutional review board waived the requirement of written informed consent for participation from the participants or the participants’ legal guardians/next of kin because note that the data analyzed comes from Twitter – considered to be public data.

## Author contributions

EA-C and AF: conceptualization, supervision, and writing – review and editing. EA-C, MT, AF, and AK: data curation, formal analysis, and investigation. MT and AK: writing – original draft preparation. EA-C: project administration and funding acquisition. All authors contributed to the article and approved the submitted version.

## Footnotes

^1^This tweet was translated from Spanish.
